# Supplementary material for: Low BCL-xL expression in triple-negative breast cancer cells favors chemotherapy efficacy, and this effect is limited by cancer-associated fibroblasts
Source: Sci Rep. 2024 Jun 19;14:14177. doi: 10.1038/s41598-024-64696-z (PMC11187150; doi:10.1038/s41598-024-64696-z)
Supplement: Supplementary file 1 — Supplementary Information. [file 41598_2024_64696_MOESM1_ESM.pptx]

## Slide 1
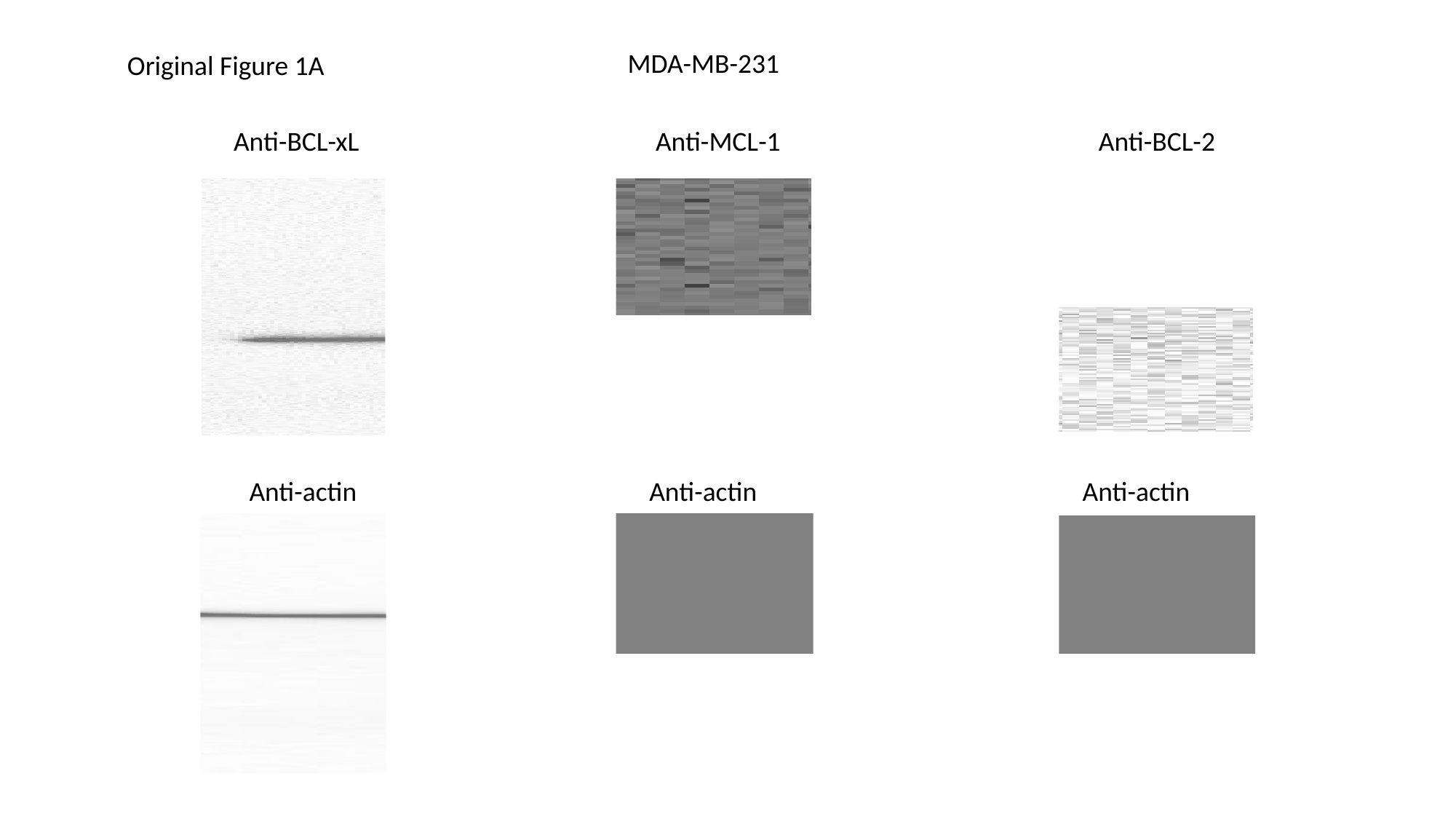

MDA-MB-231
Original Figure 1A
Anti-BCL-xL
Anti-MCL-1
Anti-BCL-2
Anti-actin
Anti-actin
Anti-actin

## Slide 2
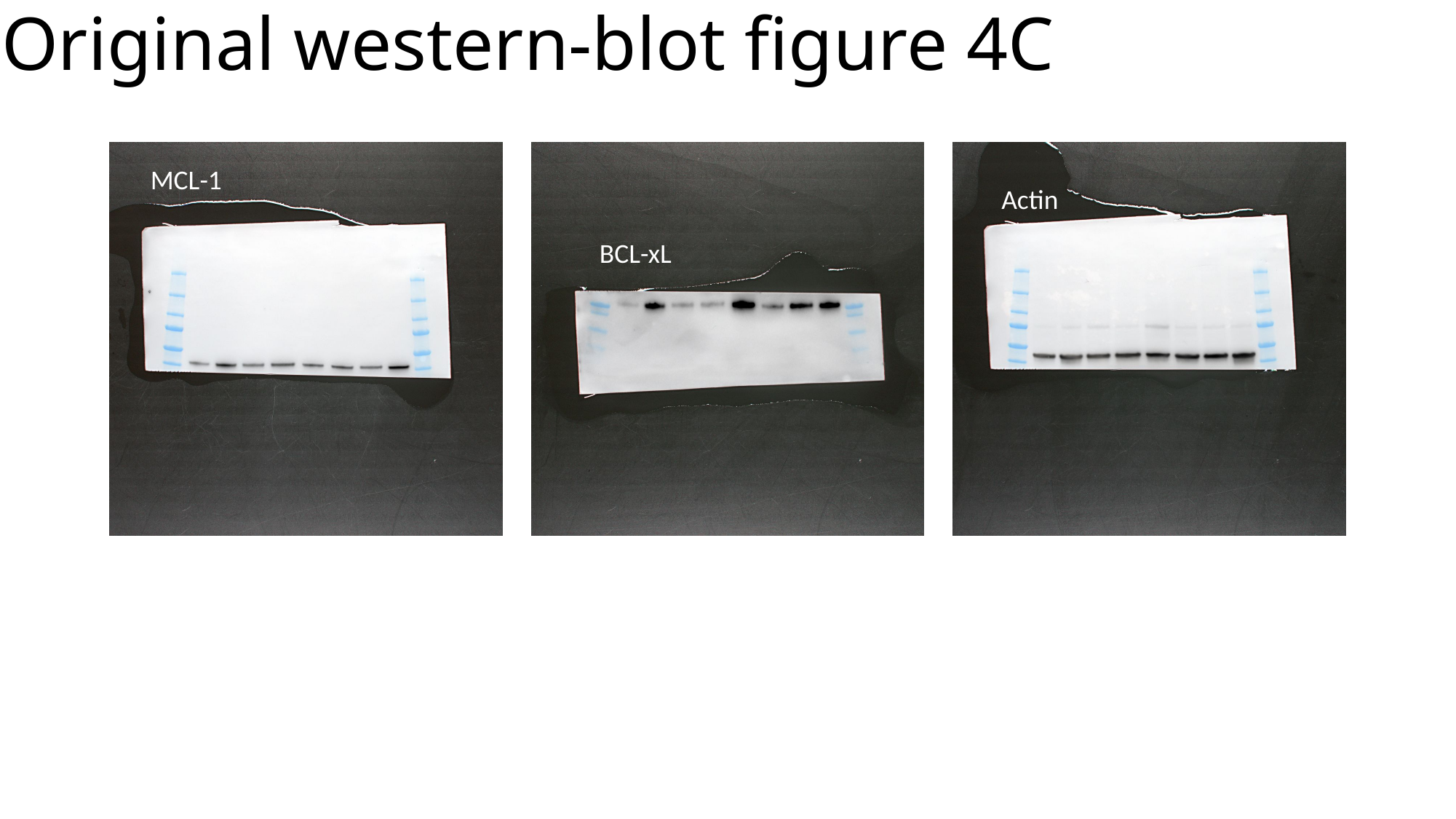

# Original western-blot figure 4C
MCL-1
Actin
BCL-xL

## Slide 3
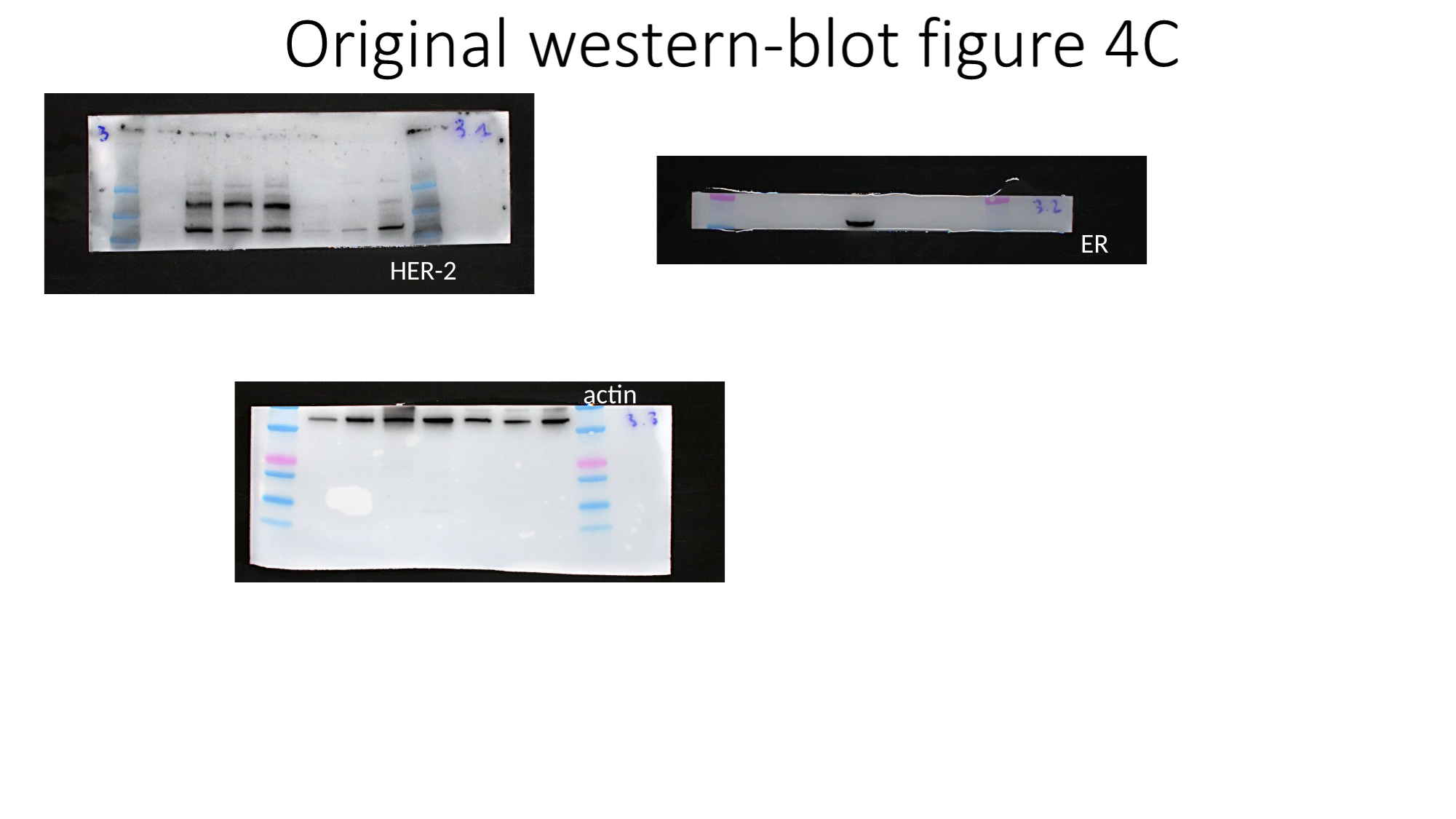

ER
HER-2
actin

## Slide 4
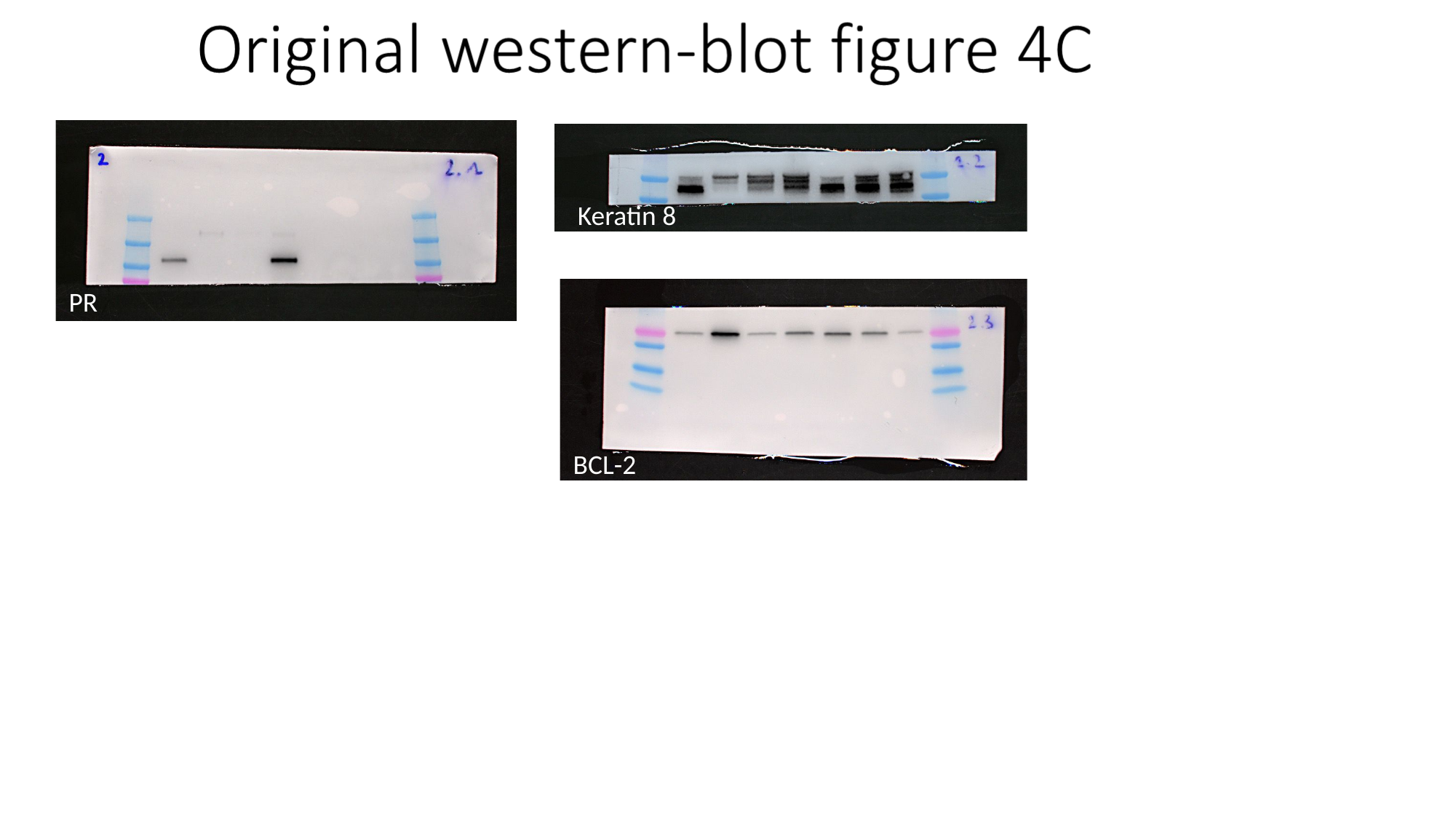

Keratin 8
PR
BCL-2

## Slide 5
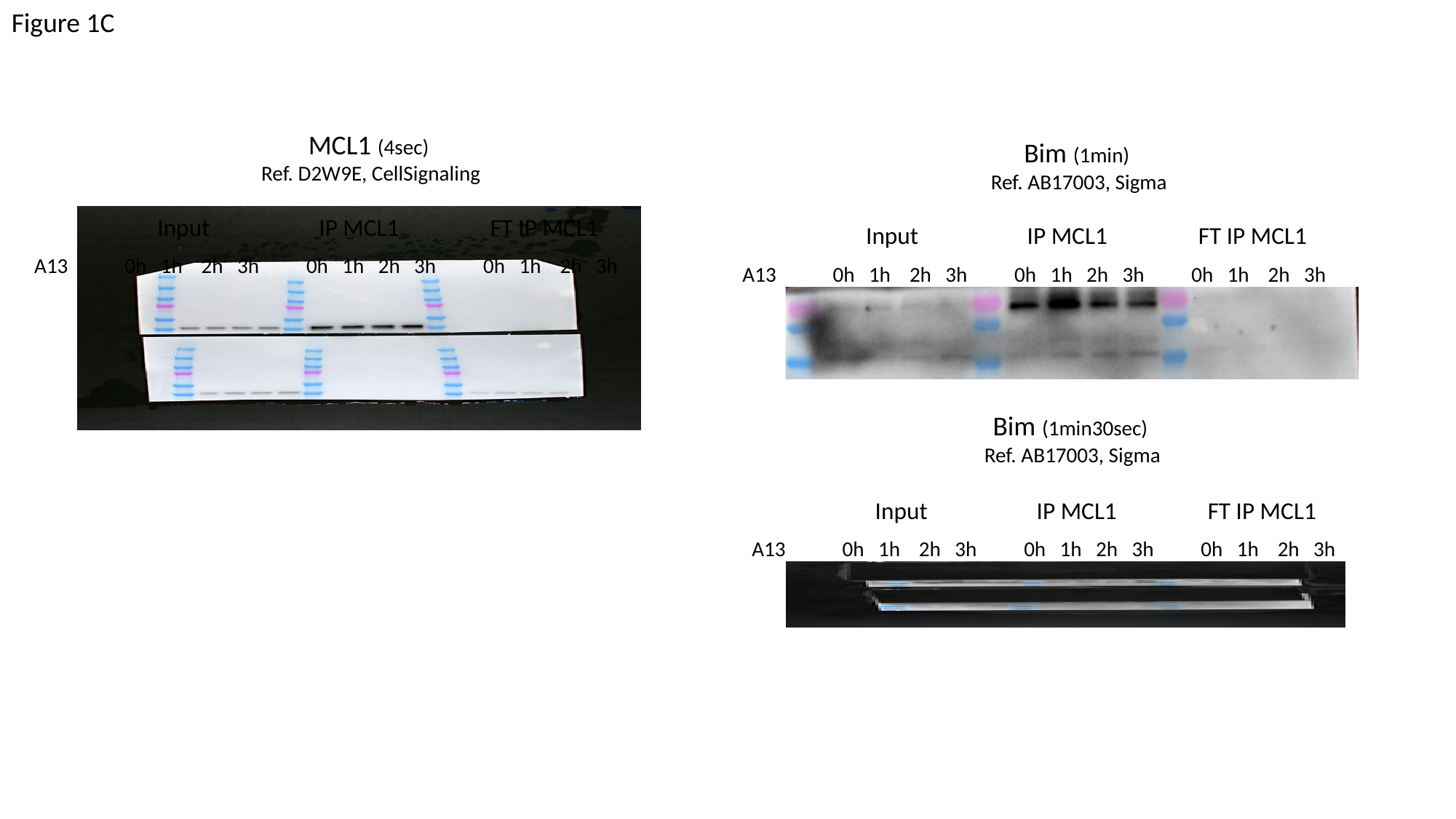

Figure 1C
MCL1 (4sec)
Ref. D2W9E, CellSignaling
Bim (1min)
Ref. AB17003, Sigma
Input
IP MCL1
FT IP MCL1
Input
IP MCL1
FT IP MCL1
A13 0h 1h 2h 3h 0h 1h 2h 3h 0h 1h 2h 3h
A13 0h 1h 2h 3h 0h 1h 2h 3h 0h 1h 2h 3h
Bim (1min30sec)
Ref. AB17003, Sigma
Input
IP MCL1
FT IP MCL1
A13 0h 1h 2h 3h 0h 1h 2h 3h 0h 1h 2h 3h
